# Genome Sequence of *Arthrobacter globiformis* B-2979 Phage MUWow

**DOI:** 10.17912/micropub.biology.002354

**Published:** 2026-10-29

**Authors:** Brian S Burnes, Robert Sample, Benjamin Onyeagucha, Jess H Collins, Philip J Evans, Kaliyah J Moore, Nykeria J Pratt, Cadie Wright

**Affiliations:** 1 Department of Sciences and Mathematics, Mississippi University for Women, Columbus, MS, USA

## Abstract

We report the discovery and genome sequence of MUWow, an AY-cluster temperate bacteriophage with siphovirus morphology. MUWow was isolated from a soil sample from Columbus, MS, USA in 2024. It has a genome size of 55,034 bp with 94 predicted protein-encoding genes, 53 of which are assigned putative functions.

**Figure 1. Genome annotation of phage MUWow:** The 3 forward reading frames are shown in light shading, the 3 reverse reading frames are shown in dark shading, and trans-membrane proteins are shown in diagonal fill. Nine phams are fully conserved across the cluster: the portal protein (pham 307765), the head -to -tail adapter (pham 337836), the helix-turn-helix DNA binding protein domain (pham 336670), the SSB protein (pham 337856), the glutaredoxin/NrdH-like glutaredoxin/thioredoxin (pham 338233), the DNA helicase/methylase (pham 336516), a second helix-turn-helix DNA binding protein domain (pham 337870), the DNA polymerase III sliding clamp beta (pham 338056), and an additional conserved hypothetical protein (pham 338065) with no known function. Data analyzed September, 2026.

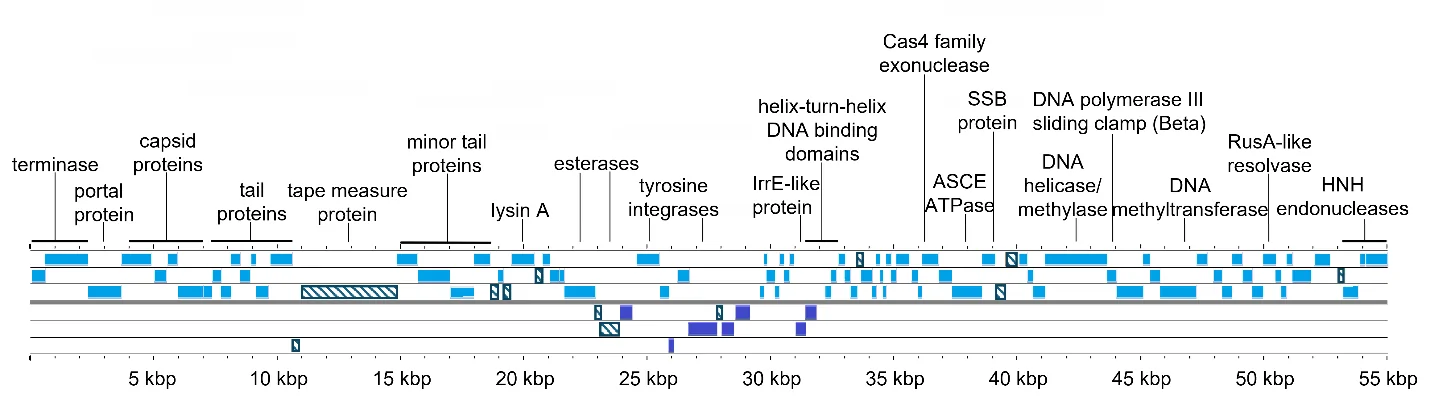



## Description

Actinobacteriophages are known to be genetically diverse, and the isolation and characterization of novel actinobacteriophages contribute to our growing understanding of co-evolutionary dynamics and the development of phage therapy applications (Pope et al, 2015; Pedulla et al, 2003; Millhaven et al., 2023; Strathdee et al., 2023). We present the genome of MUWow, a bacteriophage isolated in 2024 from a soil sample collected in Columbus, Mississippi (GPS coordinates 33.493021 N, 88.419611 W), using *Arthrobacter globiformis* B-2979 as the isolation host.

Following standard procedures (Poxleitner et al., 2018), approximately 15 cm ^3^ of soil was suspended in 35 mL PYCa medium and shaken at 37°C at 250 rpm for 2 h, followed by centrifugation at 2,000 *g* and vacuum filtration (0.22 µm filter) of the supernatant. This filtrate was inoculated with *A. globiformis* B-2979 and incubated at 30°C for 5 days at 250 rpm. An aliquot was spun at 14,000 *g* , filtered, and the filtrate plated in PYCa top agar containing *A. globiformis B-2979* . After 48 h, MUWow formed clear plaques with defined edges 2 mm +/- 0.1 mm (n=25) in diameter and was purified through two rounds of plating.

DNA was extracted from a lysate (Wizard DNA Clean-up kit, Promega), prepared for sequencing (NEBNext Ultrall FS kit), and sequenced (Illumina NextSeq 1000, XLEAP-P1 reagents) resulting in 1,536,867 single-end 100-bp reads with 2,633-fold coverage. Raw reads were trimmed with cutadapt 4.7 (using the option: –nextseq-trim 30) and filtered with skewer 0.2.2 (using the options: -q 20 -Q 30 -n -l 50) prior to assembly (Martin, 2011; Jiang et al., 2014; Wick et al., 2017; Gordon et al., 1998). As described by Russell (Russell, 2018), the genome was assembled (Newbler v2.9) (Margulies et al., 2005) and checked for completeness and genome termini (Consed v29.0) (Gonrdon et al., 1998), resulting in a genome 55,034 bp in length with 9-bp 3' single-stranded overhang ends and a GC content of 62.4%.

Using standard procedures (Pope et al., 2017), the software DNA Master v5.23.6 (Pope et al, 2018), Genemark v2.5p (Lukashin et al., 1998), and Glimmer v3.02 (Delcher et al., 1999) were used to automatically predict 98 putative protein-encoding genes. Start sites were refined using Starterator v485 (Pacey, 2016) and Blastp v2.13.0 (Altschul et al., 1990) alignments against the Actinobacteriophage protein database (Russell and Hatfull, 2017) and NCBI non-redundant protein sequences databases (Camacho et al, 2009). No tRNA genes were found using Aragorn v1.2.41 (Laslett and Canback, 2004) and tRNAscan-SE v2.0 (Lowe and Eddy, 1997). A total of 53 genes were assigned putative functions using BLASTp v2.13.0, Phamerator (Creshawn et al, 2011), and HHPred (searching against PDB_mmCIF70, SCOPe70, Pfam-A, and NCBI_Conserved_Domains databases) (Soding et al., 2005). deepTMHMM v1.0.24 (Krogh et al., 2001) and SOSUI (Hirokawa et al., 1998) detected 12 membrane proteins. Using the gene content similarity (GCS) tool at the Actinobacteriophage database and based on GCS of at least 35% of phages in the database, MUWow is assigned to the AY cluster (Pope et al., 2017; Jacobs-Sera et al., 2020). All software used default settings. The annotated genome is presented in Fig. 1.

As with other AY phages, all predicted genes are transcribed unidirectionally with the exception of a central region of genes transcribed in the reverse direction (Adams et al, 2025). MUWow is similar to other Cluster AY genomes which are organized with genes involved in structure and assembly in the first third of the genome, followed closely by genes involved in lysis, and then by genes associated with lysogeny towards the center of the genome (Adams et al, 2025). Genes related to recombination/replication are at the beginning of the last third of the genome.

The MUWow genome (55,034 bp) is characteristic of AY phages, which range from 49,447bp to 56,481 bp. It has a GC content of 62.4%, placing it in the bottom half of Cluster AY (62.1-63.6), and 99 genes, slightly above the median in the Cluster AY range of 89 to 104 genes. Similar to nearly all other Cluster AY phage, MUWow has 9-base 3' single-stranded ends with the sequence CGCCGGTGA. It encodes 9 phams (phage gene families) that are fully conserved across the cluster: the portal protein (pham 307765), the head -to -tail adapter (pham 337836), the helix-turn-helix DNA binding protein domain (pham 336670), the SSB protein (pham 337856), the glutaredoxin/NrdH-like glutaredoxin/thioredoxin (pham 338233), the DNA helicase/methylase (pham 336516), a second helix-turn-helix DNA binding protein domain (pham 337870), the DNA polymerase III sliding clamp beta (pham 338056), and an additional conserved hypothetical protein (pham 338065) with no known function (Data analyzed September, 2026). MUWow has two transmembrane proteins flanking its endolysin and a putative holin. The conservation of these flanking genes and its proximity to the lysin and holin genes suggests a role in phage lysis. All phages in the cluster encode genes associated with lysogeny, including 2 tyrosine integrases, one in the forward direction and the second downstream in the reverse direction. Downstream of these integrases, MUWow also contains a gene that is conserved across AY phages and has, in some instances, been functionally assigned as an excise. The presence of both tyrosine integrases and excise suggests MUWow may be temperate, which is consistent with experimental data demonstrating lysogen formation for other cluster AY phages (Hussain et al, 2025; Boyle, 2026).

**Data availability**

MUWow is available at GenBank under the accession No. **PX307277** and at the Sequence Read Archive (SRA) under **SRR35152158 .**
